# Cigarette Smoke Enhances the Expression of Profibrotic Molecules in Alveolar Epithelial Cells

**DOI:** 10.1371/journal.pone.0150383

**Published:** 2016-03-02

**Authors:** Marco Checa, James S. Hagood, Rafael Velazquez-Cruz, Victor Ruiz, Carolina García-De-Alba, Claudia Rangel-Escareño, Francisco Urrea, Carina Becerril, Martha Montaño, Semiramis García-Trejo, José Cisneros Lira, Arnoldo Aquino-Gálvez, Annie Pardo, Moisés Selman

**Affiliations:** 1 Instituto Nacional de Enfermedades Respiratorias, Ismael Cosío Villegas, Ciudad de México, México; 2 Instituto Nacional de Medicina Genómica, Ciudad de México, México; 3 Department of Pediatrics, Division of Respiratory Medicine, University of California San Diego, and Rady Children's Hospital of San Diego, San Diego, California, United States of America; 4 Facultad de Ciencias, Universidad Nacional Autónoma de México, Ciudad de México, México; Helmholtz Zentrum München, GERMANY

## Abstract

Idiopathic pulmonary fibrosis (IPF) is a progressive and lethal disease of unknown etiology. A growing body of evidence indicates that it may result from an aberrant activation of alveolar epithelium, which induces the expansion of the fibroblast population, their differentiation to myofibroblasts and the excessive accumulation of extracellular matrix. The mechanisms that activate the alveolar epithelium are unknown, but several studies indicate that smoking is the main environmental risk factor for the development of IPF. In this study we explored the effect of cigarette smoke on the gene expression profile and signaling pathways in alveolar epithelial cells. Lung epithelial cell line from human (A549), was exposed to cigarette smoke extract (CSE) for 1, 3, and 5 weeks at 1, 5 and 10% and gene expression was evaluated by complete transcriptome microarrays. Signaling networks were analyzed with the Ingenuity Pathway Analysis software. At 5 weeks of exposure, alveolar epithelial cells acquired a fibroblast-like phenotype. At this time, gene expression profile revealed a significant increase of more than 1000 genes and deregulation of canonical signaling pathways such as TGF-β and Wnt. Several profibrotic genes involved in EMT were over-expressed, and incomplete EMT was observed in these cells, and corroborated in mouse (MLE-12) and rat (RLE-6TN) epithelial cells. The secretion of activated TGF-β1 increased in cells exposed to cigarette smoke, which decreased when the integrin alpha v gene was silenced. These findings suggest that the exposure of alveolar epithelial cells to CSE induces the expression and release of a variety of profibrotic genes, and the activation of TGF-β1, which may explain at least partially, the increased risk of developing IPF in smokers.

## Introduction

Idiopathic pulmonary fibrosis (IPF) is a chronic, progressive, irreversible and lethal disease with a median survival of 3 years after diagnosis [1]. Multiple pathogenic mechanisms have been hypothesized, but recent evidence indicates that there is a repetitive injury to the alveolar epithelial cells, which, in susceptible individuals, may cause their aberrant activation and the exaggerated release of a variety of profibrotic mediators including TGF- β1 [2, 3].

TGF-β1 plays a critical role in IPF because it is a key activator of fibroblast to myofibroblast differentiation and regulates numerous genes involved in the synthesis and accumulation of extracellular matrix and in the disordered wound healing that characterizes this disease [4].

Furthermore, TGF-β1 activated myofibroblasts in IPF lungs induce alveolar epithelial cell death and cause breakdown of basement membranes, which contributes to the failure of reepithelialization [5,6]. The final result is the excessive deposition of extracellular matrix and the destruction of lung parenchyma architecture [1].

Although the etiology of IPF is unknown, several epidemiological studies have found that smoking is a major risk factor in patients with both sporadic and familial disease [7,8,9,10,11,12]. Furthermore, studies in experimental models of pulmonary fibrosis support this evidence [13,14,15]. For instance, we have shown that guinea pigs instilled with bleomycin and exposed to cigarette smoke display a significant increase in the number of myofibroblasts and in the extent of fibrotic lesions compared with guinea pigs that had received bleomycin alone [16].

However, the molecular mechanisms by which cigarette smoke increases the risk of IPF have not been elucidated. The aim of this study was to evaluate the effect of cigarette smoke on the gene expression profile in alveolar epithelial cells, focusing on the expression of profibrotic genes and in the regulation of signaling pathways likely involved in the pathogenesis of IPF.

## Materials and Methods

Official Mexican Standard and the Guide for the Care and Use of Laboratory Animals of the National Research Council. Ethics committee of Instituto Nacional de Enfermedades Respiratorias approved this study. Pentobarbital was used for animal sacrifice.

### Cell Culture

Human alveolar epithelial cell line (A549) was obtained from ATCC (CCL-185). The cells were cultured in DMEM with 10% fetal bovine serum (GIBCO Laboratories, Grand Island, NY), penicillin [100 U/ml] and streptomycin [100 mg/ml] at 37°C in a gas mixture of 5% CO_2_ / 95% air in 25 cm^2^ culture flasks (T-25; Corning, Costar). After reaching early confluence the cells were trypsinized and plated for experiments.

Lung epithelial cell lines from mice (MLE-12; CRL-2110) and rat (RLE-6TN; CRL-2300) were purchased from ATCC (Manassas, VA). MLE-12 cells were cultured in DMEM: F-12 medium with insulin (5 ng/ml), transferrin (0.01 mg/ml), sodium selenite (30 nM, hydrocortisone (10 nM), B-estradiol (10 nM), HEPES 10 nM, glutamine and 10% FBS (GIBCO Laboratories, Grand Island, NY). RLE-6TN cells were grown in Ham's F12 medium supplemented with bovine pituitary extract (0.01 mg/ml), insulin (5 ng/ml), insulin-like growth factor (2.5 ng/ml), transferrin (1.25 μg/ml), EGF (2.5 ng/ml), and 10% SFB (GIBCO Laboratories, Grand Island, NY).

### Preparation of cigarette smoke extract (CSE)

The cigarette smoke extract was prepared using a modification of the method developed by Aoshiba [17]. Cigarettes (Marlboro, Philip Morris) were subjected to unfiltered combustion using a 60ml syringe. The cigarette smoke was bubbled into 25 ml of culture medium (DMEM-F12 without serum); the resulting suspension was adjusted to pH 7.4 and filtered with a 0.2 μM membrane. This solution was considered 100% and CSE was applied in a period no longer than 20 minutes after preparation.

### Exposure of epithelial cells to CSE

Cell cultures at 80% confluence were left in culture medium without serum 24 hours before the start of the experiment followed by incubation with CSE 1%, 5% and 10% in DMEM plus 1% of FBS (fetal bovine serum); CSE media was refreshed every 24 hrs. The cell cultures were maintained for a week, and sub-cultured each time at a density of 6.0 x 10^6^ cells per 100mm dish, until completing 3 and 5 weeks of stimuli respectively.

### Growth rate assay

A549 cells were exposure with CSE 5% or 10% for 1 week, re-plated in 96-well culture plates at a density of 1.5 × 10^4^ cells/well and incubated at 37°C in 5% CO_2_-95% air. After 24 hrs, cell growth was examined by the WST-1 assay according to the manufacturer instructions (Roche Applies Science, Indianapolis, IN). Absorbance was analyzed on an ELISA plate reader at 450 nm, with a reference wavelength of 620 nm.

### Cell viability

A549 cells were cultured for 1 week in 6-well flat-bottom plates at 9.0 x 10^5^ cells per well in the presence of DMEM with 1% fetal bovine serum (control), or plus CSE at 5% or 10%. Cells were trypsinized, combined (1:1) with Trypan blue reagent (T10282; Invitrogen), transferred onto a Countess Cell Counting Chamber Slides (C10313; Invitrogen) and subjected to automated assessment using the Countess automated cell counter (C10227, Invitrogen).

### Microarray analysis

RNA from human alveolar epithelial cells exposed to CSE and controls was extracted with Trizol (Invitrogen Life Technologies, Grand Island, NY) and was hybridized to Whole-Transcript Microarrays (Human Gene 1.0 ST, Affymetrix, Santa Clara, CA) according to the Affymetrix protocol.

Microarray data was pre-processed using R and Bioconductor. Raw intensity values were background corrected, log2 transformed and then RMA normalized [18], using algorithms coded in the “oligo” package in Bioconductor. To identify differentially expressed genes we fit a linear model using the limma package [19]. Finally, lists of differentially expressed genes for each of the three comparisons were created selecting those genes that are statistically significant according to two summary statistics: the log fold-change and the B-statistic. The cutoff for the fold-change was chosen to be equal to 1 combined with a confidence measure based on the B-statistic greater than zero. The microarray analysis was performed in 3 individual experiments.

The data discussed in this publication have been deposited in NCBI's Gene Expression Omnibus and are accessible through GEO Series accession number GSE77942. (http://www.ncbi.nlm.nih.gov/geo/query/acc.cgi?acc=GSE77942).

### Ingenuity Pathway Analysis (IPA)

From the list of differentially expressed genes between the two groups, canonical pathways and related functions were studied using Ingenuity Pathway Analysis (IPA) bioinformatic software, identifying networks differentially regulated between the two groups (p value <0.05).

### RNA extraction and real-time PCR

Messenger RNA was obtained using the TRIZOL reagent and 1 μg of RNA was reverse transcribed into complementary DNA (cDNA) (Advantage RT-for-PCR Kit, Clontech, Palo Alto, CA) according to the manufacturer instructions.

Step One Real-Time PCR System (Applied Biosystems, CA) was used for amplification by real-time PCR, using FAM-labeled Taqman assays (Human probes: Hs01548727_m1 for MMP-2, Hs01042796_m1 for MMP-7, Hs00234244_m1 for TGFβ2, Hs00998133_m1 for TGFβ1, Hs00983056_m1 for neural cadherin (CDH2), Hs01549976_m1 for fibronectin, Hs00185584_m1 for vimentin, Hs00234140_m1 for monocyte chemoattractant protein (CCL2), Hs00233808_m1 for integrin alpha V (ITGαV) (Hs00899660_g1), and Hs00234579_m1 for MMP-9. Mouse integrin alpha V probe: Mm00434486_m1, rat integrin alpha V probe: Rn01485633_m1. 18S Ribosomal RNA (Eukaryotic 18S rRNA endogenous Control) was used as housekeeping gene. All PCRs were carried out in a mixture of 25 μl, which contained 4 μl of cDNA and 12.5 μl of 2X PCR Master Mix (Applied Biosystems). The PCR conditions were 2 min at 94° C followed by 40 cycles of 15 s at 94° C and 60° C 1 m. Results from three different experiments performed in triplicate are expressed as mean ± SD of 2 ^ -ΔCT of target gene normalized to 18S rRNA.

### Extraction and Quantification of Proteins

RIPA lysis buffer (Santa Cruz) plus a cocktail of serine and cysteine protease inhibitors (Calbiochem) was used for the extraction of proteins in cell lysates. Protein concentration was determined by Bradford Assay Reagent (Bio-Rad Laboratories Inc., Hercules, CA).

### Western Blot

The levels of ITGαV (Santa Cruz. Cat. Sc-10729; dilution 1:200), fibronectin (Santa Cruz. Cat. Sc-80205; dilution 1:1000), vimentin (Santa Cruz. Cat. Sc-373717; dilution 1:1000) and MMP-9 (Santa Cruz Cat. SC-21733; dilution 1:200) were measured in A549 lysates by immunoblotting using denaturing polyacrylamide gels at 12%. The same proteins were measured in MLE-12 and RLE-6TN lysates: vimentin (Abcam, Cat. Ab92547; dilution 1:200), fibronectin (Abcam, ab45688; dilution 1:200), ITGαV (Santa Cruz, Cat. sc-10729; dilution 1:200), and MMP-9 (Santa Cruz, Cat. sc-6840; dilution 1:200). Gels were transferred to nitrocellulose membrane (Osmonics, Westboroug, MA) and blocked with fat free milk at 5% in TBS/0.1% Tween. Membranes were incubated with primary antibody specific for each protein overnight. The secondary antibody was used coupled to peroxidase (anti-rabbit, anti-goat or anti-mouse, Zymed, CA). Proteins were visualized by chemiluminescence (Pierce, USA) and normalized against beta-tubulin. (Biolegend Cat. 622102).

### ELISA

CCL-2 and TGF-β concentrations were evaluated in cell culture supernatants by ELISA commercial kits from R & D Systems, Inc. (DCP00 and DB100B respectively) according to the manufacturer instructions. TGF-β was also quantified in bronchoalveolar lavage (BAL) from rats exposed to cigarette smoke. 200 μl of cell culture supernatant or BAL were incubated for 2 hrs at room temperature. After washing 4 four times with 200 μl wash buffer conjugated CCL-2 was added and incubated for 1 hr at room temperature. Following washes, 200 μl of substrate solution was added and after 30 minutes the reaction was stopped. The plate was read in a spectrophotometer at 450 nm.

### Animal model

The experimental model was performed in 8 week-old male Wistar rats randomly separated in experimental and controls. The animals were placing into the whole-body chamber and they were fed ad libitum with a diet of croquettes and sterile water, with cycles of light/darkness for 12 hours and constant temperature as previously reported [20].

The animals were exposed to tobacco smoke for two months in an acrylic chamber (70 cm long x 50 cm wide x 30 cm in height) with 8 mm diameter hole in opposite sides. In one of the holes was placed the burning cigarette without filter and on the opposite side a rubber tube connected to a vacuum pump (Rocker 600, 1/4 HP, air flow 60 l/min; Rocker Scientific Co., Ltd., Taiwan) so that the smoke will circulate continuously inside the chamber. Groups of 5 rats were exposed for 30 min to smoke of 5 cigarettes and after that the camera was opened to release the smoke and the animals were maintained in regular room air for another 30 min. The procedure was repeated 4 times a day until complete 20 cigarettes. Five non-exposed animals were used as controls. The animals were treated according to the specifications for the care and use of laboratory animals of the Official Mexican Standard and the Guide for the Care and Use of Laboratory Animals of the National Research Council. The animals were killed under anesthesia with pentobarbital (Pisebantal, PiSA México) at 50 mg/kg.

Lungs from animals were lavaged twice through a tracheal cannula by instilling 5-ml aliquots of sterile PBS solution at 37°C. BAL fluids were centrifuged at 400 g for 10 min at 4°C and the supernatants were kept at -80°C.

### Histological Analysis

The lungs were washed with saline via the pulmonary artery and infiltrated with 4% paraformaldehyde at a constant pressure of 25 cm H_2_O. Tissue sections (6 mm) were embedded in paraffin, sectioned at 4 μm with a microtome (Rotary Histostat Reichet) and the sections were permanently mounted using a synthetic resin. The sections were observed and photographed using a microscope Carl Zeiss and digital images were captured using Zeiss AxioCam MRC5 camera.

### Immunohistochemistry

Tissues from rats exposed to tobacco smoke and controls were deparaffinized, and endogenous peroxidase activity was blocked. Subsequently, antigen retrieval with citrate buffer (10 mM, pH 6,0) was performed in a microwave. Slides were blocked for 20 min with 1x universal blocker (BioGenex) and the samples were incubated with anti ITGαV antibody (Santa Cruz. Cat.Sc-10729) 1:200. Finally, the sections were incubated for 20 minutes with an antibody coupled to peroxidase (BioGenex) and aminoethylcarbazole was used as substrate.

### Stable silencing ITG αV

A549 cells (7.5x10^5^) were plated in 12-well plates, with F12 and 10% FBS. Viral infection was performed using 75,000 lentiviral particles containing short hairpin RNA (shRNA) targeting ITGαV (Santa Cruz Biotechnology, Texas) and a mixture of polybrene to a final concentration of 5 mg/mL. The infection efficiency was evaluated using a positive control of lentiviral particles with GFP (green fluorescent protein). The infected cell clones were obtained from 10, 100 and 1000 cells in each well by serial dilutions and stable clones were selected via Puromycin at a concentration of 7.5 μg/mL. Resistant colonies were expanded in 6-well plates. As a negative control we used plasmid-A (sc-108060), encoding a scrambled shRNA sequence that will not lead to the specific degradation of any cellular message, also obtained from Santa Cruz Biotechnology.

### Wound healing assay

A549 cells were incubated with medium DMEM +1% FBS (controls) or plus cigarette smoke extract (CSE 5% and 10%) for 3 weeks. Then, the cells were seeded at a density of 9.0 x 10^5^ in sterile 6-well plates and a wound was made using a pipette tip (200 μl). Additionally, 2.5 μg/ml mitomycin C (Sigma M4287) was added to each well to inhibit proliferation. Cell migration to close the wound was monitored at 72 hours and was photographed with a digital camera mounted on an inverted microscope (Olympus). The area was calculated with the ImageJ software (imagej.nih.gov/ij/) drawing a line along the edge of the wound. Assays were done in three independent experiments.

### Statistical Analysis

All data were expressed as mean ± SD of 3 or 4 determinations. P values were calculated by using one-way ANOVA test. P values <0.05 were considered statistically significant.

## Results

### CSE induces incomplete epithelial to mesenchymal transition

**Fig 1:**
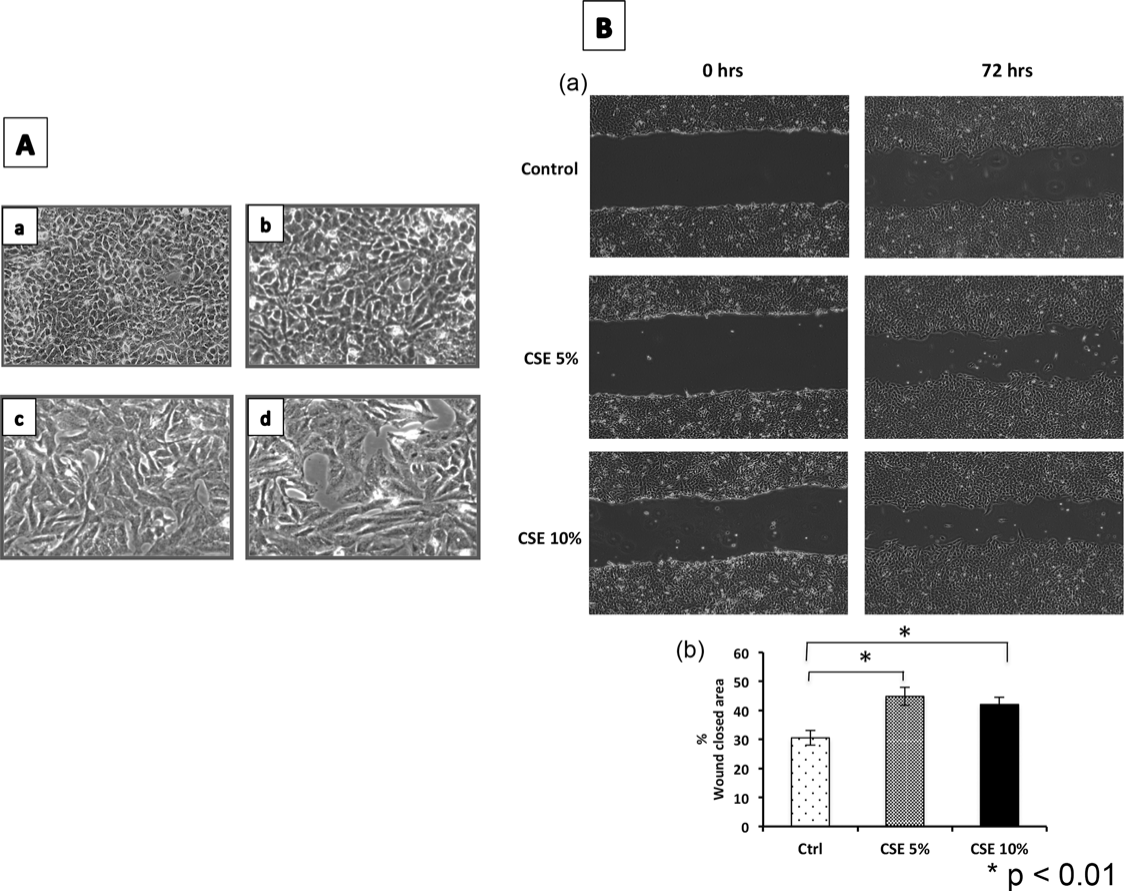
Exposure to CSE induces morphological changes in epithelial cells with a higher migratory capacity. **(A)** A549 exposed for 5 weeks to different concentrations of cigarette smoke extract. (a) Control, (b) CSE 1%, (c) CSE 5%, (d) CSE 10%. **(B) (a)** A549 cells showed faster wound closure at 72 hours in the presence of 5 and 10% of CSE; **(b)** The percent of closed wound area was calculated versus the initial time of each condition with the ImageJ software. Values represent the mean ± SD obtained from three different experiments. *p<0.01 versus untreated controls.

Human lung epithelial cells (A549), exposed to three different concentrations of cigarette smoke extracts (1%, 5% and 10%) during 1, 3 and 5 weeks were studied. From the third week of exposure, but mainly at 5 weeks, epithelial cells exposed to CSE at concentrations of 5% and 10% displayed morphologic changes acquiring an elongated, fibroblast-like shape (Fig 1A). They also showed a higher migratory capacity compared with untreated cells (Fig 1B) suggesting that the cells underwent a process of epithelial to mesenchymal transition (EMT). Similar results were observed with mouse (MLE-12) and rat (RLE-6TN) epithelial cells (not shown). Cigarette smoke extracts did not affect cell viability (S1A Fig) although a slight but significant decrease in cell proliferation was observed with 10% of CSE (S1B Fig).

**Fig 2:**
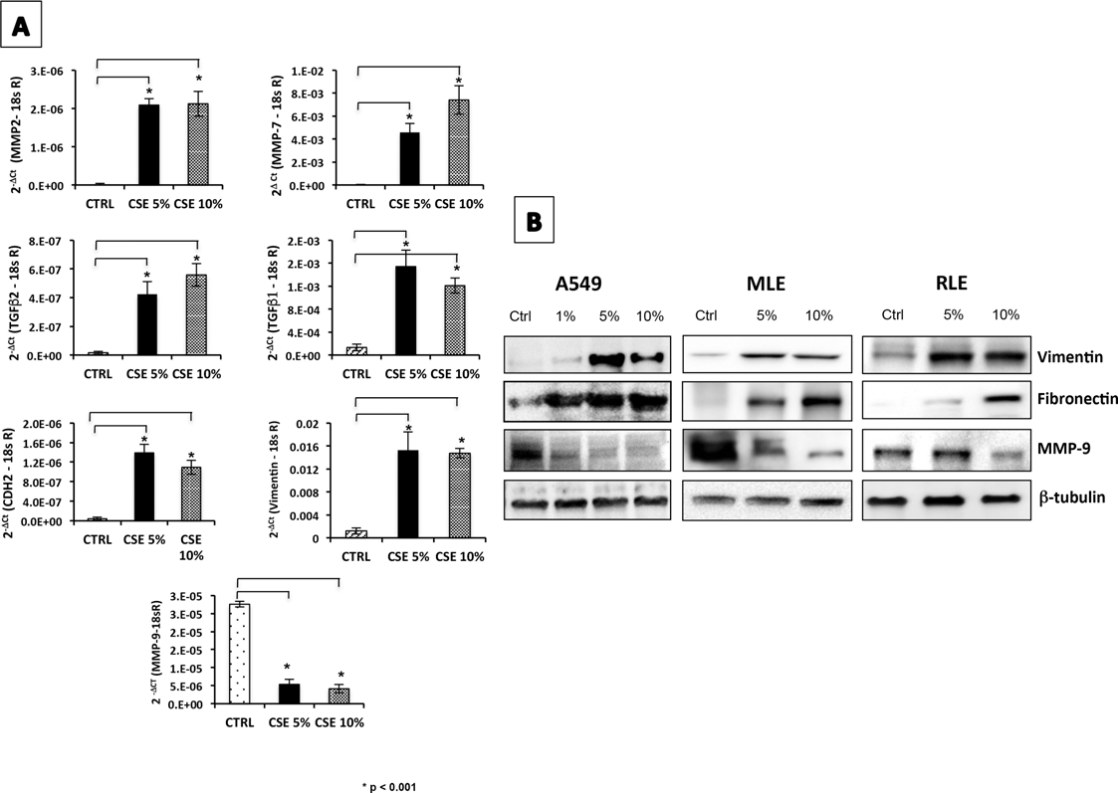
Lung epithelial cells exposed to CSE express genes and produce proteins related to fibroblast phenotype. **(A)** The levels of mRNA expression were measured by real-time PCR in A549 cells after 5 weeks of exposure to CSE (5% and 10%). The expression levels of all genes were normalized to 18sRNA. Ctrl = Control cells, CSE 5% = A549 exposed to 5% cigarette smoke extract, CSE 10% = A549 exposed to 10% CSE. Values represent the mean ± SD obtained from three different experiments performed in triplicate. *p <0.001 versus untreated control. **(B)** After 5 weeks of exposure, levels of EMT-related proteins (vimentin, fibronectin and MMP-9) were measured by Western blotting in cell lysates of A549, MLE-12 and RLE-6TN. b-tubulin was used as endogenous protein. Blots are representative of two independent experiments.

Gene expression evaluated by microarrays in A549 cells (see below), showed that a number of genes likely related to EMT were dysregulated. For example, vimentin, CDH2, MMP-2 and fibronectin were over-expressed, while MMP-9 (gene that is not usually expressed by lung fibroblasts) was decreased in response to long-term culture in CSE. Selected differentially expressed genes in A549 cells (vimentin, MMP-2, MMP-7, CDH2, MMP-9, TGF-β1 and TGF-β2) were validated by real-time PCR (Fig 2A). Likewise, the increase of vimentin and fibronectin and the decrease of MMP-9 provoked by CSE were corroborated at the protein level in A549, MLE-12 and RLF-6TN lysates by Western blot (Fig 2B). However, decrease of E-cadherin (characteristic of loss of epithelial identity) was not found, suggesting that the exposure to CSE in alveolar epithelial cells causes only a partial epithelial to mesenchymal transition.

### Microarray analysis

**Fig 3:**
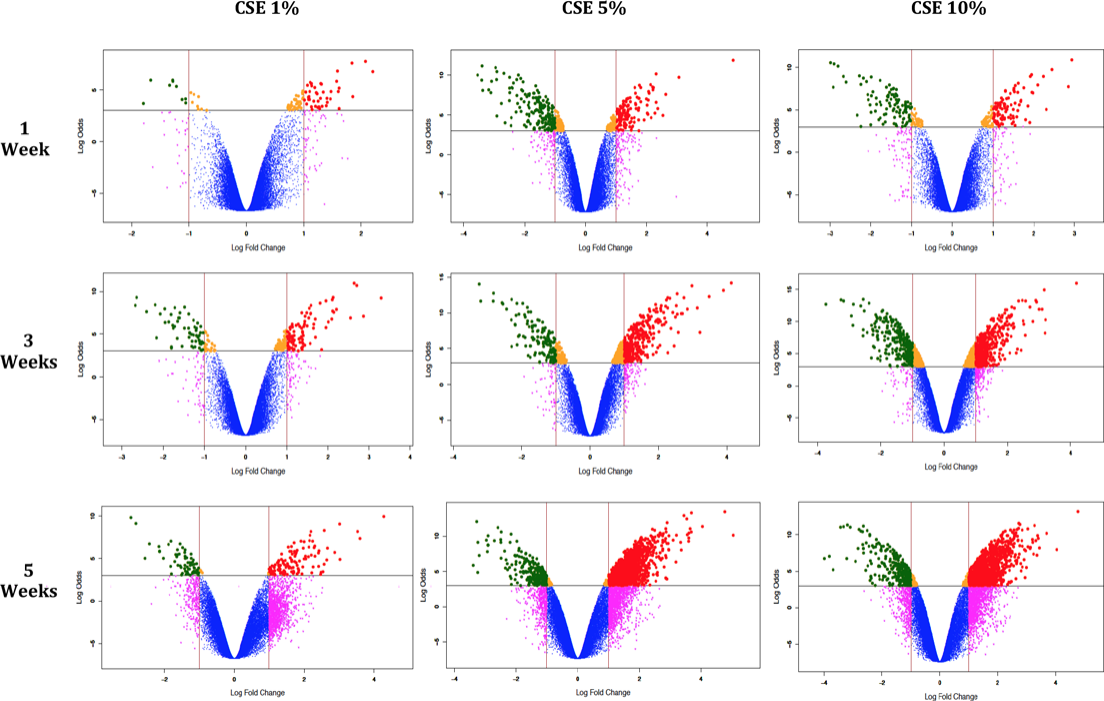
Volcano plots of genes differentially expressed in A549 cells stimulated with CSE 1%, 5% and 10% versus control cells. The x-axis represents the value of the log fold change (logarithm base 2) between different experimental conditions. The y-axis represents the B value (log-odds). Genes up-regulated > 1-fold with a B-value >3 are depicted in the red dots and those down regulated with identical fold change and B-value are indicated in green dots.

**Fig 4:**
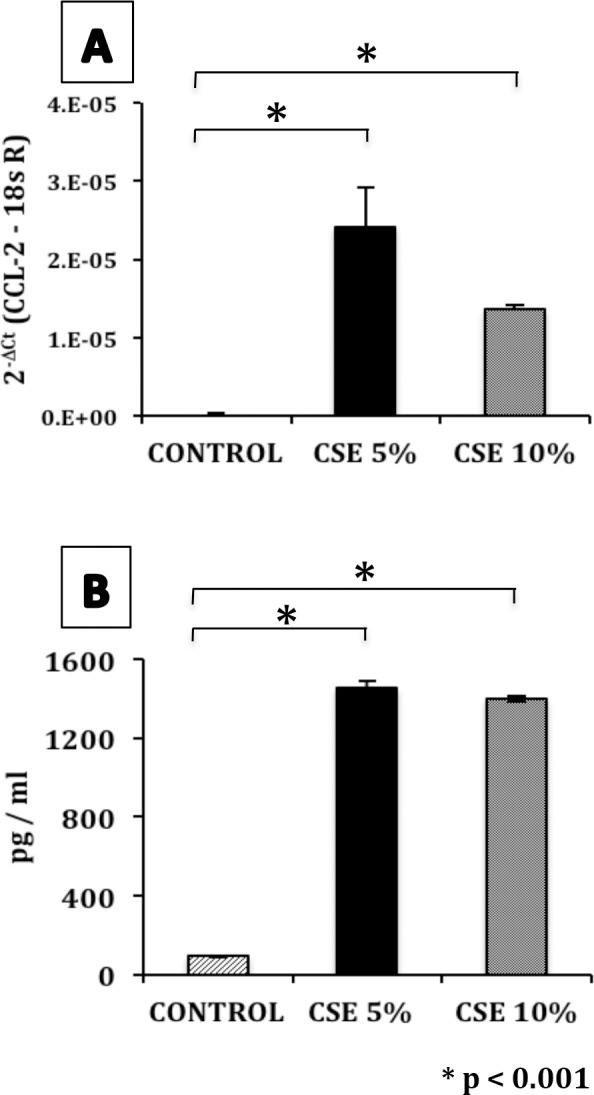
CSE induces the expression and production of CCL-2 in A549 cells. **(A)** After 5 weeks of exposure to CSE 5% or 10%, CCL-2 mRNA expression was measured by real-time PCR. The CCL-2 expression levels were normalized to 18sRNA. Values represent the mean ± SD obtained from three different experiments performed in triplicate. * p <0.001 versus untreated control. **(B)** The levels of CCL-2 in supernatants were examined by ELISA in cells exposed for 5 weeks to CSE 5% or 10%. Values represent the mean ± SD obtained from four different experiments performed in triplicate. CTRL = A549 unstimulated, CSE 5% = A549 exposed to CSE 5%, CSE 10% = A549 exposed to CSE 10%. *p <0.001 versus untreated control.

Differential expression analysis was performed by Whole-Transcript Array (Human Gene ST 1.0, Affymetrix, Santa Clara CA), in three independent experiments that included three different exposure times (1, 3 and 5 weeks), and different CSE concentrations (1%, 5% and 10%). As seen in the volcano plots of Fig 3, CSE epithelial stimulation caused a progressive increase in the numbers of genes overexpressed, based on exposure time and concentration. After 5 weeks of exposure, over 1000 upregulated genes were detected in cells exposed to the CSE compared to the control group. The top 100 deregulated genes are shown in Table 1. Of these genes, the chemokine CCL-2 showed the highest expression. CCL-2 among other functions induces migration of fibroblasts, fibrocytes and lymphocytes to sites of injury and has been associated with the pathogenesis of IPF [21,22,23]. This chemokine was upregulated from the first to the fifth week of exposure and its over-expression was validated at the mRNA and protein levels by qPCR (Fig 4A) and ELISA respectively (Fig 4B).

**Table 1:** 

sym	logFC	P.Value	sym	logFC	P.Value
CCL2	5.03	2.1E-08	STEAP1	2.60	5.4E-08
CYP1B1	4.76	2.8E-10	MIR224	2.59	4.4E-06
CD302	4.05	4.8E-09	NR4A1	-2.59	5.9E-09
FN1	3.68	3.6E-10	IL6ST	2.55	5.6E-07
PLEKHH2	3.68	1.2E-08	BIRC3	2.55	3.2E-07
QPCT	3.64	7.1E-09	BICC1	2.54	2.4E-06
ST851A4	3.60	1.6E-08	TMOD2	2.54	6.3E-08
NR5A2	3.59	5.5E-08	IFNGR1	2.53	1.4E-05
HPGD	3.55	2.0E-08	ATRX	2.52	1.4E-07
CDH6	3.53	1.2E-09	ST3GAL5	2.51	2.7E-08
MAP2	3.44	6.1E-10	ZNF518A	2.51	1.6E-06
AKT3	3.33	6.5E-08	C3	2.50	6.4E-08
TFF1	-3.27	2.1E-09	PXDN	2.49	9.8E-09
FGB	-3.24	4.5E-06	AADAC	2.49	1.3E-07
MAPK4	-3.23	6.2E-08	MSLN	-2.49	4.1E-06
TM4SF18	3.20	2.0E-08	NR1D1	-2.48	6.3E-08
MMP7	3.18	1.1E-06	TBC1D19	2.44	3.1E-08
TMEM156	3.09	4.5E-08	LPCAT2	2.44	5.9E-06
FGA	-2.96	4.6E-08	MANEA	2.31	1.9E-07
DIAPH2	2.96	2.4E-08	TFPI	2.31	1.6E-06
AMIG02	2.95	2.0E-07	ZNF644	2.31	7.7E-08
CEP170	2.95	1.9E-07	SLC6A14	2.30	1.3E-06
NCOA7	2.93	7.8E-08	CEP170	2.29	4.8E-06
FSTL4	-2.91	2.3E-08	ARL4C	2.28	7.8E-08
SLFN5	2.90	2.5E-09	IGF2	2.28	7.1E-06
ATRX	2.90	l.lOE-05	ARID4A	2.28	4.5E-07
STAT4	2.90	7.4E-08	BMPR1B	2.26	3.8E-08
HIPK2	2.87	1.2E-08	ATP8B1	2.26	1.4E-07
CFHR1	2.87	6.0E-07	TGFB2	2.26	1.6E-07
IL8	2.86	7.2E-09	TSPAN7	2.25	6.7E-08
MLLT11	2.86	3.5E-08	VPS13C	2.08	l.ZOE-06
ARHGAP18	2.85	4.3E-07	TIA1	2.08	6.5E-06
F2RL2	2.84	5.5E-08	ZBTB20	2.07	9.3E-07
GPR126	2.81	2.1E-06	KLRC3	2.07	1.3E-06
ITGB6	2.78	1.6E-08	CYorf15B	2.07	2.0E-06
CFH	2.76	4.8E-08	GLIPR1	2.07	2.3E-07
STEAP2	2.76	6.3E-08	RAB11FIP2	2.07	3.6E-07
TIPARP	2.75	2.3E-08	KLHL5	2.06	1.3E-06
CLEC4E	2.75	6.3E-07	ITGAV	2.00	3.0E-06
TGFB1	2.73	2.9E-06	PCM1	1.99	5.2E-07
NID2	-2.71	5.1E-08	TTC3	1.99	1.2E-05
GCC2	2.70	8.1E-07	FU36840	1.99	2.0E-05
NTRK3	2.68	2.0E-08	KIAA1107	1.99	5.5E-07
MIR31	2.68	2.9E-07	SPOPL	1.99	7.6E-05
NEDD4	2.68	1.5E-08	ZBTB41	1.99	1.2E-06
RP56KA6	2.68	1.5E-08	SEMA3C	1.99	1.6E-07
APOH	2.66	6.5E-07	HRSP12	1.99	1.9E-05
D5T	2.64	2.4E-08	AP152	1.98	3.6E-06
IFNE	2.63	5.1E-07	RIF1	1.98	2.1E-06
PLA2R1	2.61	2.0E-08	CPB2	1.98	1.1E-07

### TGF- β is a possible modulator of phenotypic changes in epithelial cells exposed to CSE

**Fig 5:**
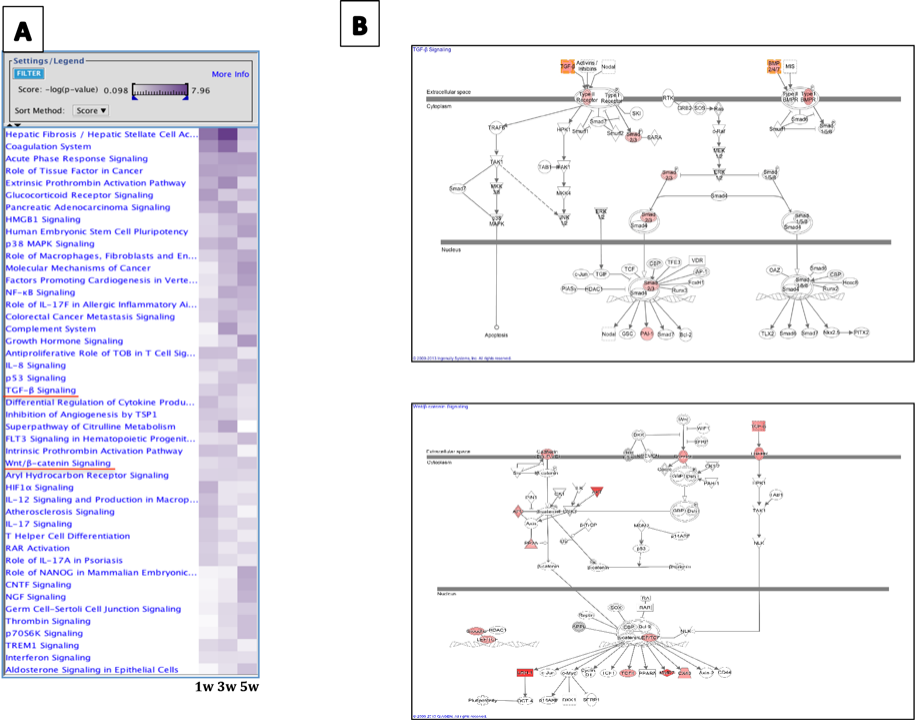
**(A)** Main canonical pathways induced by cigarette smoke extract at 1, 3 and 5 weeks of exposure. 1w (1 week of exposure to 5% CSE), 3w (3 weeks of exposure to 5% CSE), 5w (5 weeks of exposure to 5% CSE). **(B)** Deregulated genes (red) in the TGF-β1 signaling pathway (upper panel) and Wnt signaling pathway (bottom panel) in epithelial cells exposed to cigarette smoke extract compared to control cells.

Network pathways were constructed using the IPA bioinformatic software. Among the main dysregulated pathways were TGF-β and Wnt signaling (Fig 5A). Thus, genes such as TGFβ1, TGFβ2, the type II receptor for TGF-β, Smad2 and 3, and plasminogen-activator inhibitor 1 (PAI-1) were found over-expressed in these CSE-exposed cells, as well as the Wnt receptor Frizzled (Fig 5B).

**Fig 6:**
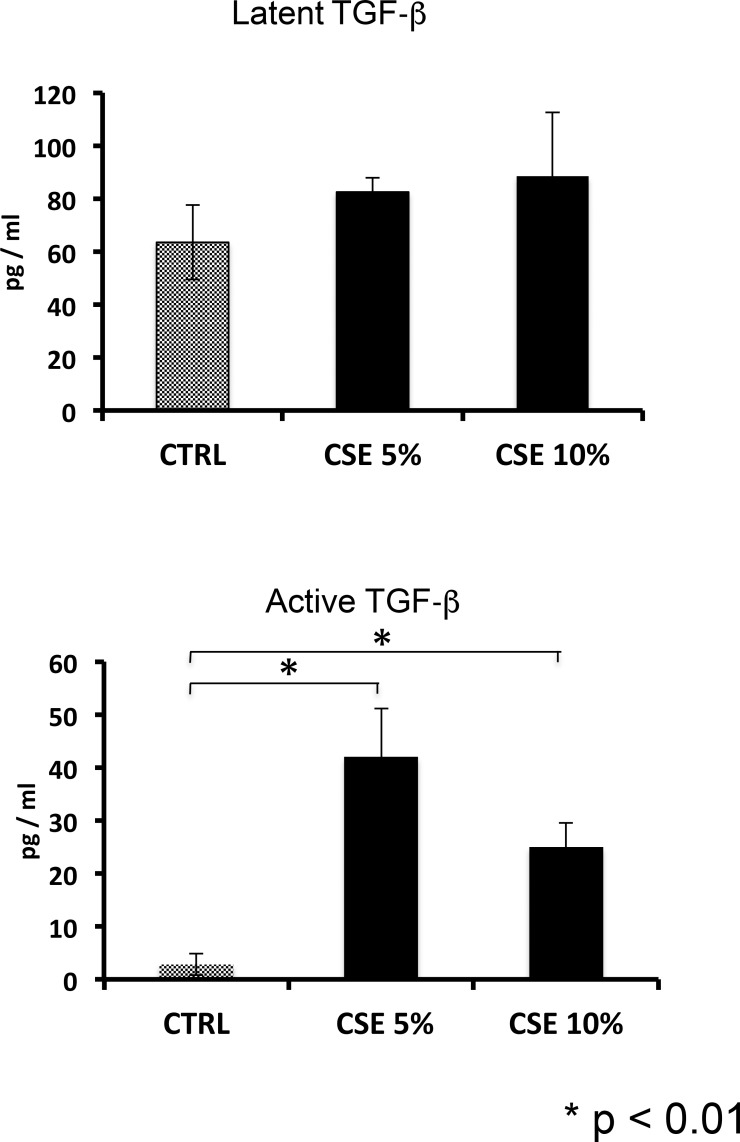
CSE promotes the activation of TGF-β. After one week of stimulation, the levels of latent and active TGF-β1 were examined by ELISA in supernatants of epithelial cells exposed to cigarette smoke. CTRL = A549 unstimulated, CSE 5% = A549 exposed to CSE 5%, CSE 10% = A549 exposed to CSE 10%. Results from four independent experiments each of them in triplicate are expressed as mean +SD. * p <0.01 versus untreated control.

After validating the gene expression levels of TGF-β1 by real-time PCR (Fig 2A), we examined the levels of total TGF-β (latent and active) protein secreted into the conditioned media from cells exposed to CSE. No significant differences of latent TGF-β1 were found between the cells exposed to cigarette smoke and the control cells. By contrast, the levels of active TGF-β were significantly increased in cells exposed to cigarette smoke (Fig 6).

### Integrin αV β6 mediates latent TGF- β activation in stimulated epithelial cells

To explore the mechanism(s) involved in the activation of latent TGF-β, the expression of several genes known to be implicated in this process were examined from our transcriptional profiling. We found that the gene levels of αV (ITGαV) and β6 integrins (ITGβ6) were increased. The heterodimer of this integrin is an important activator of latent TGF-β [24].

**Fig 7:**
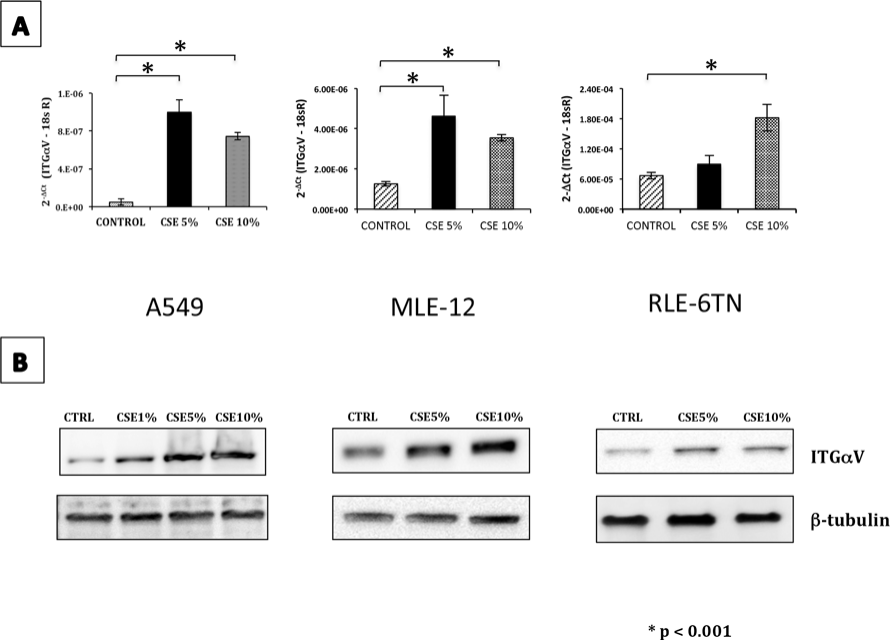
Exposure to CSE increases the expression and production of ITGαV in lung epithelial cells. **(A)** mRNA levels of ITGαV were quantified by real-time PCR in A549, MLE-12 and RLE-6TN cells after one week of exposure to CSE; data were adjusted to 18sRNA levels. Values represent the mean ± SD obtained from three different experiments performed in triplicate. *P<0.001 versus untreated control. **(B)** Quantification of cell lysates of ITGαV by Western blotting in A549, MLE-12 and RLE-6TN cells after one week of exposure. CTRL (A549 unstimulated), CSE 1% (A549 exposed to CSE 1%), CSE 5% (A549 exposed to CSE 5%), CSE 10% (A549 exposed to CSE 10%).

**Fig 8:**
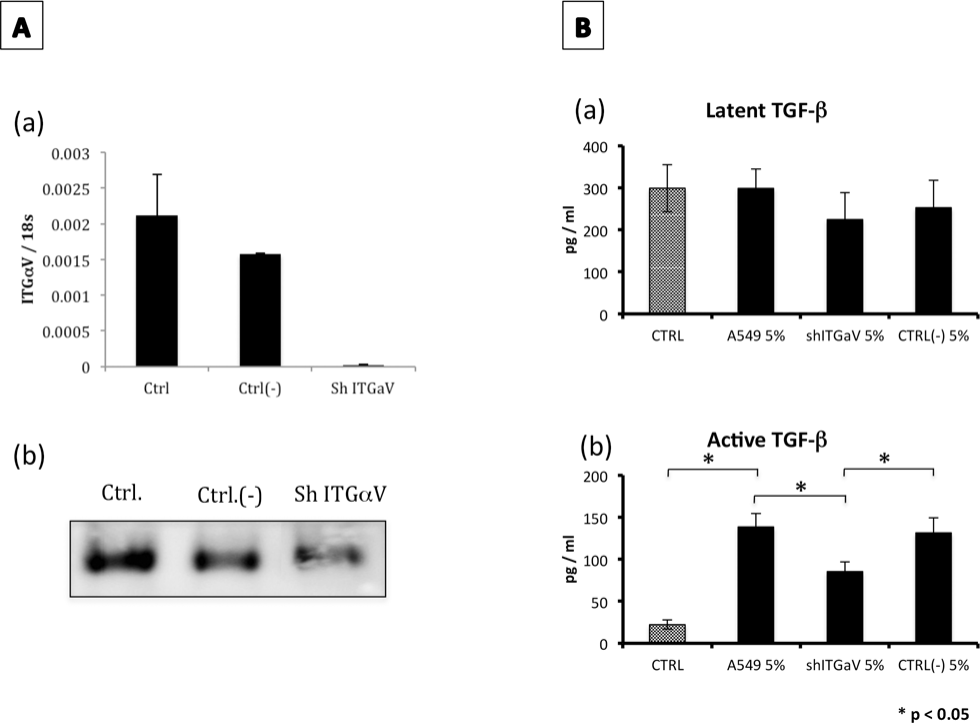
ITGαV-silenced cells exposed to CSE showed a partial decrease in TGF-β activation. **(A)** The expression of ITGαV was measured at mRNA level by real-time PCR (panel a). Values represent the mean ± SD obtained from three different experiments performed in triplicate (panel b) Quantification of ITGαV in cell lysates by Western blotting. **(B)** After one week of exposure to CSE 5%, the levels of latent (panel a) and active (panel b) forms of TGF-β were examined in the conditioned media by ELISA. Values represent the mean ± SD obtained from four different experiments performed in triplicate. *P<0.05. Ctrl (unstimulated A549), A549 5% (A549 exposed to 5% CSE), shITGαV 5% (A549 infected with shITGαV exposed to 5% CSE), Ctrl (-) 5% (A549 infected with a scrambled shRNA sequence exposed to 5% CSE). *p <0.05.

The upregulation of integrin αV was corroborated in A549, MLE-12 and RLE-6TN cell lines at both mRNA (Fig 7A) and protein levels (Fig 7B). As a proof of concept, the αV integrin was silenced using viral vector encoding specific short-hairpin RNA. The expression of ITGαV was completely abolished at the mRNA level and partly at the protein level (Fig 8A).

After 1 week of stimulation with CSE 5%, TGF-β concentration was quantified by ELISA in control and in ITGαV silenced cells. No significant differences were observed with latent TGF-β (Fig 8B, upper panel). As expected, control epithelial cells (either normal or infected with the empty vector) stimulated with 5% cigarette smoke extract showed a higher concentration of active TGF-β compared with non-stimulated cells. The ITGαV gene silenced cells showed a partial but significant decrease in TGF-β activation. (Fig 8B, bottom panel).

**Fig 9:**
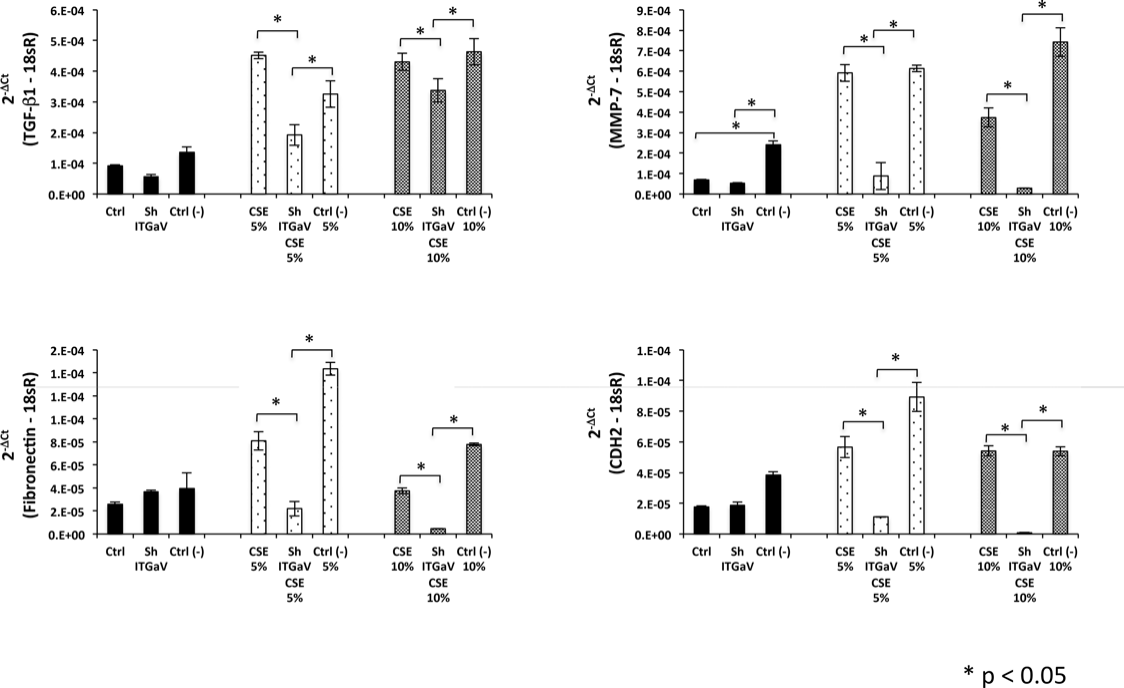
Knockdown of ITGαV inhibits the expression of EMT-related genes in A549 exposed to CSE. The levels of mRNA expression of TGF-β1, fibronectin, CDH-2 and MMP-7 were measured by real-time PCR in A549 cells after 1 week of exposure to CSE (5% and 10%). The expression levels were normalized to 18sRNA. **Black bars:** Ctrl (unstimulated A549 cells), shITGαV (A549 infected with shITGαV) and Ctrl (-) (A549 infected with a scrambled shRNA sequence). **Dotted bars:** CSE 5% (A549 exposed to CSE 5%), shITGαV 5% (A549 infected with shITGαV exposed to CSE 5%) and Ctrl (-) 5% (A549 infected with a scrambled shRNA sequence exposed to CSE 5%). **Gray bars:** CSE 10% (A549 exposed to CSE 10%), shITGαV 10% (A549 infected with shITGαV exposed to CSE 10%) and Ctrl (-) 10% (A549 infected with a scrambled shRNA sequence exposed to CSE 10%). Data represent mean +SD from three independent experiments each in triplicate. *P<0.05.

To confirm that activation mediated by ITGαV is involved in the partial EMT observed in CSE-treated cells, several EMT-related genes like TGF-β1, MMP-7, fibronectin and CDH2 were measured in the ITGαV-silenced cells exposed to CSE. ITGαV-silencing caused a down-regulation of fibronectin, CDH6, and MMP-7 compared with controls or cells infected with scrambled shRNA sequence (Fig 9). Curiously, scrambled shRNA induced a modest but significant increase of MMP-7 in controls cells.

**Fig 10:**
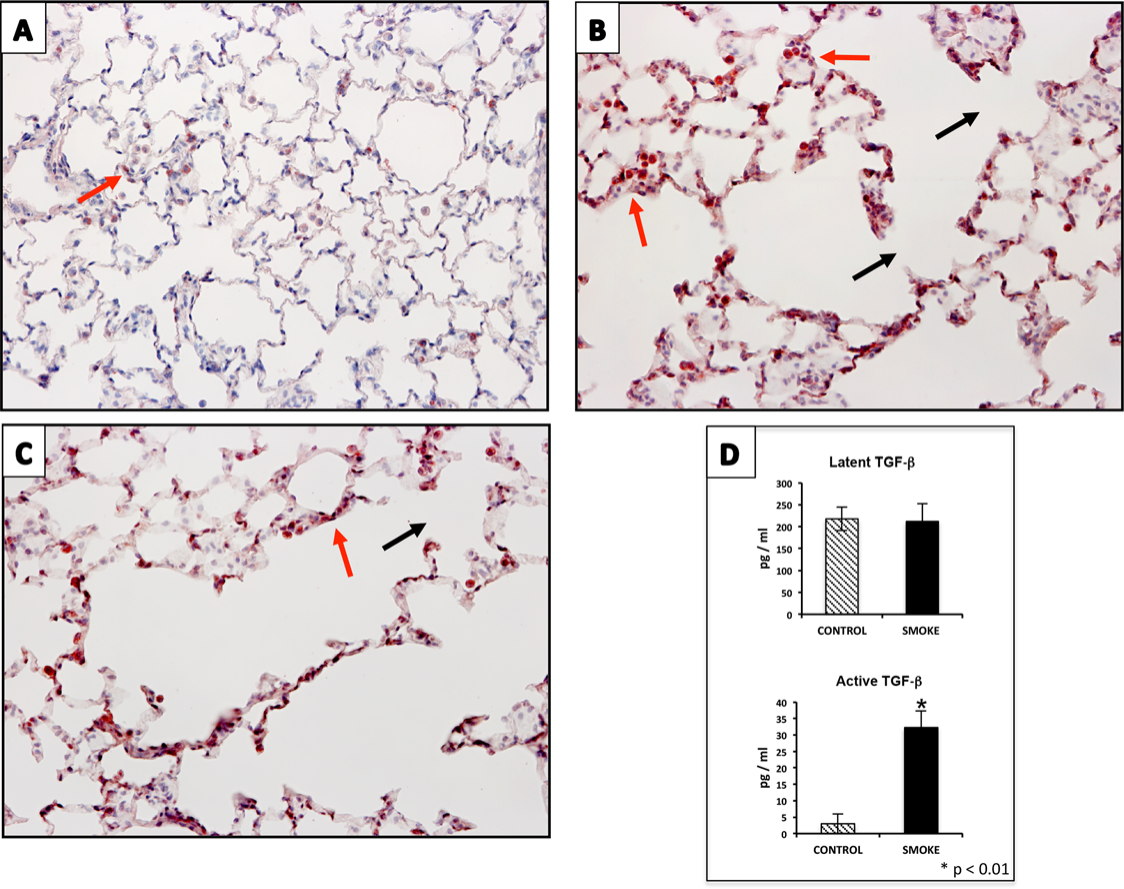
An increase in the expression of ITGαV and of active TGF-β is observed in lungs of rats exposed to cigarette smoke. Immunohistochemistry of ITGαV in control rat lung (**A; 20x**) and rat lungs exposed to cigarette smoke for 2 months (**B and C; 20x**)**.** Black arrows indicate areas of alveolar septa disruption. Red arrows highlight positive stained cells. **D)** After two months of exposure to cigarette smoke, the levels of latent and active TGF- β were examined by ELISA in BAL fluids. CONTROL = Non-treated rats. SMOKE = Rats exposed to cigarette smoke. Results from four independent experiments each of them in triplicate are expressed as mean + SD. * p <0.01 versus untreated control.

To explore whether overexpression of this integrin occurs in vivo in response to smoking, we developed an experimental model in rats exposed to cigarette smoke for two months. We observed an abundant expression of immunoreactive ITGαV after tobacco smoke exposure compared to control rats, primarily located to alveolar and bronchiolar epithelia (Fig 10A–10C). In addition, a significant increase of active TGF-β was observed in BAL fluids from rats exposed to cigarette smoke compared to non-smoker animals (Fig 10D).

## Discussion

Idiopathic pulmonary fibrosis is characterized by aberrant activation of the bronchioloalveolar epithelium and the expansion and subsequent activation of fibroblasts and myofibroblasts. Mesenchymal cells, (primarily myofibroblasts) secrete excessive amounts of extracellular matrix, mainly fibrillar collagens, leading to a progressive and irreversible destruction of the lung parenchyma [25]. However, the mechanisms that trigger the epithelial "hyperactivation" are unclear.

IPF is a complex and multifactorial disease, with a number of genetic and environmental factors contributing to disease susceptibility. Recent genome-wide association studies performed in large cohorts have revealed multiple loci that affect the epithelial integrity and the innate immune response increasing the risk of developing IPF [26].

Regarding environmental factors, exposure to cigarette smoke has been identified as the strongest risk condition associated with the development of this disease. Thus, several clinical and epidemiological studies clearly indicate that IPF, either sporadic or familial, is significantly more common in smokers [7,8,9,10,11,12]. However, the molecular mechanisms by which smoking contribute to the fibrotic response have not been elucidated.

In this context, we explored the effect of cigarette smoke extracts on the behavior of lung epithelial cells in vitro.

Our results showed that CSE activates a strong response in alveolar epithelial cells, reflected in the up-regulation of numerous genes involved in fibrosis, and morphological changes suggestive of partial epithelial-mesenchymal transition. Thus, epithelial cells exposed to different concentrations of CSE (particularly 5 to 10%) and for different times (particularly to 5 weeks) changed their phenotype to an elongated, fibroblast-like shape with increased migratory capacity and upregulation in the expression of numerous genes characteristic of fibroblasts. This is an important process since an EMT-like program changes the epithelial phenotype giving them a unique and migratory mesenchymal-like cell phenotype, and under certain circumstances may even contribute to the local expansion of fibroblast population [25]. Our results are supported by recent reports showing that short-term exposure (48 h) to cigarette smoke extracts has an effect on lung epithelial cells inducing the expression of mesenchymal markers and this process is likely mediated by the transcription factor hypoxia-inducible factor-1 alpha [27]. However, in our experimental conditions, we did not find changes in the expression level of mesenchymal markers or morphological changes suggestive of EMT in such a short period of exposure. Moreover, the overexpression of integrins, which contribute to TGF-β1 activation, was observed after a prolonged exposure period. In another study it was shown that short-term exposure with CSE provokes activation of TGFβ pathway and EMT only when epithelial cells are co-cultured with fibroblasts [28].

In our study, the observed changes in epithelial cells exposed to cigarette smoke extract were likely associated with the activation of the TGF-β signaling pathway since several components of this pathway (e.g. TGF-β1, TGF-β2 and one of its receptors), and some genes involved in its activation were over-expressed likely resulting in the increased secretion of the activated form of this factor. Importantly, activation of TGF-β is the rate-limiting step of TGF-β bioavailability [29]. We focused on αVβ6 integrin, which is an epithelial cell-restricted integrin that is expressed at low levels in normal lung but highly increased in IPF [30]. This integrin interacts with the latency-associated protein (LAP) of the inactive complexes of TGF-β1 promoting the activation of this growth factor [24]. We found that cigarette smoke exposure increases the expression of V integrin at the gene and protein level. Furthermore, our findings obtained by silencing the expression of the integrin ITGαV further indicates that TGF-β activation was at least partially mediated by this integrin complex. This observation is also supported by our in vivo experiments in rats exposed to cigarette smoke for two months, in which the lungs of exposed rats displayed an increase in the expression of ITGαV, mainly located in bronchiolar and alveolar epithelial cells along with an increase in the levels of active TGF-β.

Activated TGF-β plays a central role in the pathogenesis of IPF, primarily by promoting the activation and differentiation of fibroblasts to higher collagen-producing myofibroblasts.

Another important pathway that was activated in epithelial cells exposed to cigarette smoke extract was Wnt. In this context, a growing body of evidence has revealed that several Wnt components are up-regulated in IPF lungs, mainly in epithelial cells and fibroblasts [31]. Among other effects, Wnt signaling enhances alveolar epithelial to mesenchymal transition as well as the proliferation, activation, and resistance to apoptosis of fibroblasts [32,33].

Besides the mentioned pathways, epithelial cells exposed to CSE over-expressed many other putative profibrotic genes, including the chemokine CCL-2. This molecule is increased in pulmonary fibrosis and induces the migration of fibroblasts and fibrocytes (fibroblast progenitors) to sites of injury, which can contribute to the perpetuation of the fibrotic response [21]. Thus, various studies have shown that the expression of CCL-2 is markedly increased in IPF and in various models of pulmonary fibrosis and that the neutralization of this chemokine or its receptor significantly attenuated fibrotic response [22,23].

Our study has some limitations. First, it was performed with cell lines instead primary cultures. However, primary alveolar epithelial cells are difficult to purify and more important these cells cannot be successfully passaged for multiple times, a requirement for our long-term model. Also, we used CSE at 5% concentration that did not affect viability or proliferation, and of 10% that did not influence viability but decreased cell growth. Nevertheless, our results indicate that exposure of alveolar epithelial cells to cigarette smoke components, in the conditions used in this study, exerts a profound change in gene expression and secretion of proteins, many of which are strongly associated with the pathogenesis of idiopathic pulmonary fibrosis. These findings provide better understanding of why this disease is significantly more common in smokers and suggest likely mechanisms involving the aberrant activation of the alveolar epithelium.

## Supporting Information

S1 Fig **A) Effect of CSE exposure on cell viability.** Cell viability was measured using trypan blue staining (0.4%, Invitrogen, T10282) in A549 after one week of exposure with 5% and 10% CSE. Cells were counted by using the Countess automated cell counter (Invitrogen, C10227). Results from three independent experiments each of them in triplicate are expressed as mean ±SD. **B) Effect of CSE exposure on cell growth.** Cell growth was examined using the WST1 assay after 1 week of cigarette smoke exposure. A decrease in cell proliferation was observed with 10% of CSE. *P<0.05 versus untreated control and CSE 5%. Results from three independent experiments each of them in triplicate are expressed as mean ±SD.
(TIF)
