# Supplementary figures and images for: Cigarette Smoke Enhances the Expression of Profibrotic Molecules in Alveolar Epithelial Cells
Source: PLoS One. 2016 Mar 2;11(3):e0150383. doi: 10.1371/journal.pone.0150383 (PMC4775036; doi:10.1371/journal.pone.0150383)

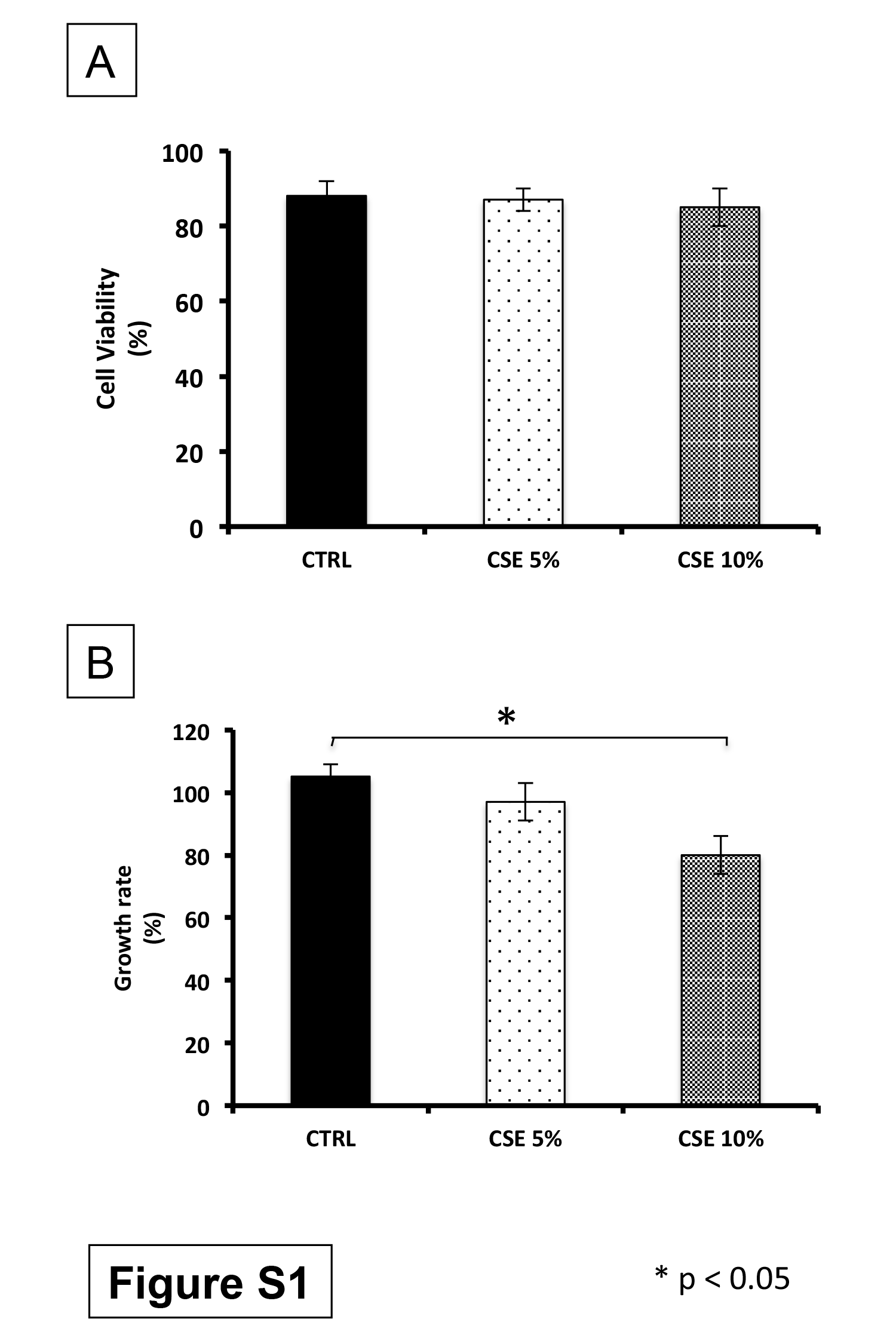

Supplement: S1 Fig — A) Effect of CSE exposure on cell viability. Cell viability was measured using trypan blue staining (0.4%, Invitrogen, T10282) in A549 after one week of exposure with 5% and 10% CSE. Cells were counted by using the Countess automated cell counter (Invitrogen, C10227). Results from three independent experiments each of them in triplicate are expressed as mean ±SD. B) Effect of CSE exposure on cell growth. Cell growth was examined using the WST1 assay after 1 week of cigarette smoke exposure. A decrease in cell proliferation was observed with 10% of CSE. *P<0.05 versus untreated control and CSE 5%. Results from three independent experiments each of them in triplicate are expressed as mean ±SD. (TIF) [file pone.0150383.s001.tif]
